# Prevalence of *Toxoplasma gondii* in Domestic Rabbits (*Oryctolagus cuniculus*) in Romania

**DOI:** 10.3390/biom16040522

**Published:** 2026-04-01

**Authors:** Anamaria Ioana Paștiu, Anca-Alexandra Doboși, Adriana Györke, Isabelle Villena, Mihai Borzan, Dana Liana Pusta

**Affiliations:** 1 Department of Genetics and Hereditary Diseases, Faculty of Veterinary Medicine, University of Agricultural Sciences and Veterinary Medicine Cluj-Napoca, 400372 Cluj-Napoca, Romania; anca.dobosi@student.usamvcluj.ro (A.-A.D.); dana.pusta@usamvcluj.ro (D.L.P.); 2 Department of Parasitology and Parasitic Diseases, Faculty of Veterinary Medicine, University of Agricultural Sciences and Veterinary Medicine Cluj-Napoca, 400372 Cluj-Napoca, Romania; adriana.gyorke@usamvcluj.ro; 3 UR ESCAPE, Faculty Medecine, University of Reims Champagne-Ardenne, 51095 Reims, France; ivillena@chu-reims.fr; 4 National Reference Centre on Toxoplasmosis and Toxoplasma Biological Resource Center, CHU Reims, General Koening Street, 51092 Reims, France; 5 Department of Animal Breeding and Food Safety, Faculty of Veterinary Medicine, University of Agricultural Sciences and Veterinary Medicine Cluj-Napoca, 400372 Cluj-Napoca, Romania; mihai.borzan@usamvcluj.ro

**Keywords:** *Toxoplasma gondii*, domestic rabbits, prevalence, MAT, nested PCR, Romania

## Abstract

*Toxoplasma gondii* is a protozoan parasite with high zoonotic potential. Currently, no information is available on natural toxoplasmosis in domestic rabbits in Romania; therefore, the aim of the present study was to evaluate the seroprevalence of *T. gondii* IgG antibodies and to determine the prevalence of *T. gondii* DNA in domestic rabbits in our country. In total, 372 domestic rabbits were tested. Blood samples were obtained from 352 animals and tissue samples were obtained from 49 animals, of which 29 animals provided paired blood–tissue samples. Samples were collected from pet rabbits and from rabbits raised in household settings, hereafter referred to as farm rabbits. Sera samples were analyzed using a modified agglutination test (MAT), with a cut-off of 1:24, for anti-*T. gondii* antibody IgG-type detection, and the tissue specimens were tested by nested polymerase chain reaction (nested PCR) targeting the B1 gene for *T. gondii* DNA detection. A seropositivity of 16.5% (58/352) was obtained by MAT, while the prevalence of *T. gondii* DNA was 4.1% (2/49). The present study highlighted the presence of *T. gondii* in domestic rabbits in Romania, which suggests that rabbit meat consumption may represent a potential risk to human health and therefore warrants further attention. Moreover, to the best of our knowledge, this is the first study to report data on the prevalence of *T. gondii* in domestic rabbits from Romania.

## 1. Introduction

*Toxoplasma gondii* (Apicomplexa: Sarcocystidae) is a globally distributed zoonotic parasite of significant medical and veterinary importance, and historically, the rabbit (*Oryctolagus cuniculus*) played a central role in its discovery [1].

The life cycle of *T. gondii* is complex, involving multiple infective stages (oocysts, tachyzoites, and tissue cysts with bradyzoites) and several transmission routes [2]. Members of the family Felidae serve as the definitive hosts, whereas all terrestrial and aquatic warm-blooded animal species, including humans and birds, can act as intermediate hosts for *T. gondii*. Definitive hosts, in which the sexual phase of the life cycle occurs, are responsible for shedding oocysts into the environment. In intermediate hosts, the parasite undergoes an asexual multiplication, characterized by the proliferation of tachyzoites during acute infection and the subsequent formation of tissue cysts containing bradyzoites [3]. Transmission occurs through multiple pathways, including the ingestion of sporulated oocysts via contaminated water, fruits, or vegetables; the consumption of viable tissue cysts in raw or undercooked meat; the ingestion of tachyzoites through raw milk; and vertical transmission across the placenta and by organ transplantation [2]. In rabbits, infection with *T. gondii* occurs through the ingestion of sporulated oocysts present on contaminated plants, fruits, vegetables, or water or through congenital transmission [2,4]. Due to feeding habits, rabbits can be considered sentinels for *T. gondii* infection, representing a good and sensitive indicator for environmental contamination with *T. gondii* oocysts [5].

The primary route of human infection with *T. gondii* is the consumption of undercooked meat containing tissue cysts. Among meat-producing animals, *T. gondii* tissue cysts are most frequently detected in pigs, sheep and goats, whereas they are less commonly found in birds, rabbits and horses and only rarely found in cattle [6]. Although rabbits are considered a less common source of tissue cysts compared with major livestock species, their role should not be underestimated. Domestic and wild rabbits may contribute to human infection through the consumption of undercooked meat [7] and, importantly, act as natural intermediate hosts in the life cycle of *T. gondii*. Together with wild rodents and small birds, wild rabbits are regarded as a natural source of infection for cats, thereby sustaining environmental contamination with oocysts and indirectly increasing the risk of infection for humans and other animals [8,9].

The rising demand for meat makes *T. gondii* in rabbits epidemiologically significant [1]. Rabbit meat is valuable [10], but its consumption in Romania remains low [11]. Nonetheless, the risk of zoonotic pathogens, including *T. gondii*, cannot be ignored. It should be noted that, in backyard systems, rabbits raised for meat are typically housed in cages or simple shelters, and their diet primarily consists of grass, alfalfa, clover, hay, vegetables, fruits, and grains. In such systems, biosecurity measures are often minimal or entirely absent. Recent studies have shown that the seroprevalence of anti-*T. gondii* antibodies in women ranges from 29.1% in central Romania [12] to 38.5% in south-western Romania, with age and rural residence identified as the main risk factors [13]. Furthermore, *T. gondii* oocysts were detected in 6.5% of cat fecal samples from both rural and urban areas of central and north-western Romania [14]. The presence of cats in the vicinity of rabbits represents a significant risk factor for *T. gondii* infection [15].

*Toxoplasma gondii* is prevalent among domestic animals in Romania, with the highest seroprevalence recorded in sheep (53.5%) [16], followed by backyard pigs (46.8%) [17], horses (39%) [18], and goat kids (33.1%) [19]. Additionally, *T. gondii* DNA was detected in 26.6% of backyard pigs [17], in 11.8% of sheep abortions [16], and in 6.1% of goat kids [19], while no DNA was found in horses [18].

Originally, three genetic lineages of *T. gondii* (types I, II, and III) with differing levels of virulence were described [20]. However, at present, at least 16 haplogroups of *T. gondii* have been identified worldwide [21]. In Romania, genotype II has been identified to date in domestic animals (goat kids, backyard pigs and lambs) [16,17,19], whereas genotypes II and III have been reported in cats [14].

There is no information related to toxoplasmosis naturally occurring in domestic rabbits in Romania, and the data related to epidemiology of *T. gondii* in rabbits in Eastern Europe is scarce. Thus, the aims of this study were to estimate the seroprevalence of *T. gondii* IgG-type antibodies and the prevalence of *T. gondii* DNA in pet and farm rabbits.

## 2. Materials and Methods

### 2.1. Animals and Sampling

From June 2022 to March 2025, samples from a total number of 372 rabbits (*Oryctolagus cuniculus*) consisting of 62 pet rabbits and 310 farm rabbits reared in a traditional household farming system were taken. Rearing system, breed, sex, age, vaccination status, body condition score (BCS), season of sampling and county of origin were recorded.

Companion rabbits were presented at the Clinic of New Companion Animals, Faculty of Veterinary Medicine, Cluj-Napoca, Romania, during which blood samples were collected. From 62 pet rabbits, comprising 33 males and 27 females, the sex of two individuals could not be determined, either due to young age or missing data; 2 were juveniles (≤4 months old) and 60 were adults (>4 months old) [22]; and 25 were vaccinated against RHD (rabbit hemorrhagic disease) 1 and/or 2 and/or myxomatosis and 37 were unvaccinated. A 1–5 BCS scale was used (1/5 represents severe underweight, 3/5 represents normal weight and 5/5 represents obese) [23]. Pet rabbits were classified as 2/5 (*n* = 4), 3/5 (*n* = 51), or 4/5 (*n* = 7) based on body weight and clinical appearance at the moment of examination. Samples were collected in spring (*n* = 8), in summer (*n* = 11), in autumn (*n* = 22) and in winter (*n* = 21).

**Figure 1:**
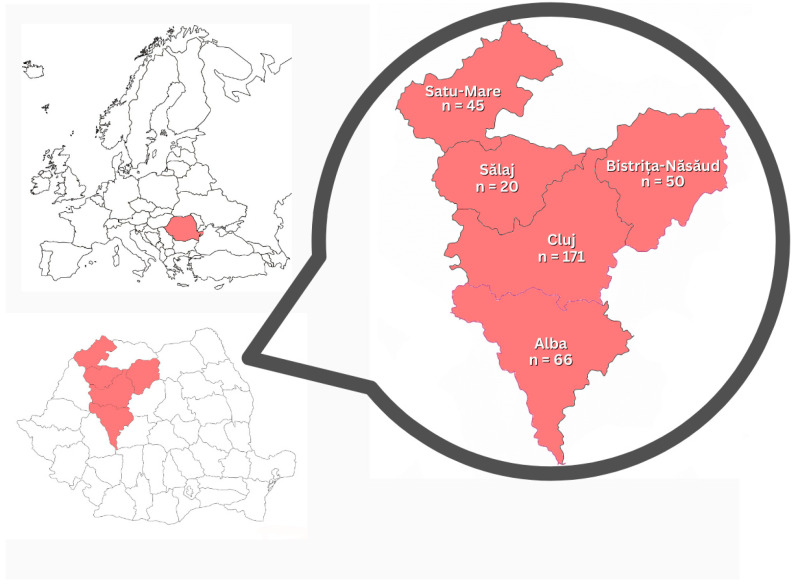
Geographical distribution of sampled rabbits (shown in red) and number of collected blood samples. Maps were taken from dmaps.com (https://d-maps.com/carte.php?num_car=25495, accessed on 10 November 2025).

Farm rabbits (*n* = 310) originated from north-western Romania (Alba, Bistrița-Năsăud, Cluj, Satu-Mare and Sălaj counties) (Figure 1). A non-probability convenience sampling approach was employed, whereby rabbits were enrolled based on the voluntary participation of consenting owners and their availability at the time of the study. No predefined selection criteria for age, sex, or clinical status were applied to the study population. The farm rabbits were reared in a small extensive system, with 1–5 rabbits per cage, depending on the age and physiological status. The study included 146 males and 157 females (the sex of seven individuals could not be determined); 69 juveniles and 241 adults; and 171 vaccinated and 139 unvaccinated rabbits. Regarding BCS, farm rabbits were classified as 2/5 (*n* = 7), 3/5 (*n* = 267), or 4/5 (*n* = 36). Samples were collected in spring (*n* = 57), in summer (*n* = 52), in autumn (*n* = 132) and in winter (*n* = 69).

The rabbit breeds and the number of animals per breed included in the present study were as follows: small breeds included Dwarf Rex (*n* = 7), Holland Lop (*n* = 13), Lionhead (*n* = 38), and Vienna Blue (*n* = 4); medium breeds included Californian (*n* = 24), Rex (*n* = 10), French Lop (*n* = 21), and Hycole (*n* = 31); large breeds included Continental Giant (*n* = 32), Flemish Giant (*n* = 23), and Transylvania Giant (*n* = 24); and the rest were mixed breeds (*n* = 145) [24].

From the 372 rabbits, blood samples and tissue specimens were collected. Blood samples (*n* = 352) were collected from the lateral saphenous vein from both pet (*n* = 48) and farm rabbits (*n* = 304), using sterile needles, following standard aseptic procedures. After collection, blood samples were allowed to clot and were subsequently centrifuged to obtain serum, which was stored at −20 °C until further serological analysis. The tissue samples, brain (*n* = 49), heart (*n* = 28), liver (*n* = 28) and lungs (*n* = 28) were obtained from 49 animals (17 pet rabbits and 32 farm rabbits) either during the necropsy examinations or slaughtering. It should be noted that both blood and tissue samples were collected from 29 rabbits. All samples were collected individually and transported to the laboratory under refrigerated conditions. Tissue samples were stored at −20 °C until molecular analysis.

The animal owners provided written consent for sample collection. This study was approved by the Animal Ethics and Welfare Committee of the University of Agricultural Sciences and Veterinary Medicine, Cluj-Napoca, Romania (No. 320/3 June 2022).

### 2.2. Modified Agglutination Test (MAT)

The modified agglutination test was performed for the detection of anti-*T. gondii* IgG type antibodies as previously described by Dubey and Desmonts [25]. Formalin-fixed whole RH strain tachyzoites were used as antigen (Reims, France). Serum samples were serially twofold diluted, starting at a 1:24 dilution (cut-off). Dilutions were performed until the endpoint, defined as the last dilution showing a positive reaction, was reached.

### 2.3. Molecular Genetics Diagnosis

DNA was extracted from the tissue samples (brain, heart, liver, and lungs) (*n* = 133) originated from 49 rabbits using DNeasy^®^ Blood & Tissue Kit (Qiagen, Hilden, Germany), following the manufacturer’s protocol.

For *T. gondii* DNA detection, nested PCR targeting the B1 gene was used, following the protocol described by Triviño-Valencia et al. [26]. Briefly, the nested PCR protocol consisted of two amplifications using the primer pairs that amplify fragments of 97 bp, namely Toxo N1 5′-GGAACTGCATCCGTTCATGAG-3′ and Toxo C1 5′-TCTTTAAAGCGTTCGTGGTC-3′ for the first PCR and Toxo N2 5′-TGCATAGGTTGCCAGTCACTG-3′ and Toxo C2 5′-GGCGACCAATCTGCGAATACACC-3′ (Generi-Biotech, Hradec Králove, Czech Republic) for the second amplification. PCR was carried out in a 25 µL reaction mixture consisting of 12.5 µL of MyTaq Red HS Mix (Meridian Bioscience, Newtown, OH, USA) and 25 pM of each primer. The volume of DNA template was 5 µL. The amplification was performed in a Bio-Rad C1000TM Thermal Cycler (Bio-Rad Laboratories, Hercules, CA, USA). The cycling conditions for the first PCR were: 5 min at 94 °C, followed by 1 min at 94 °C, 1 min at 53 °C, 1 min of extension at 72 °C (40 cycles), and a final extension for 10 min at 72 °C. For nested amplification, 2 µL of the primary PCR product was used. The second PCR protocol was: 5 min at 94 °C, followed by 1 min at 94 °C, 1 min at 53 °C, 72 °C for 30 s (14 cycles), and a final extension for 10 min at 72 °C. All PCR reactions were performed with appropriate positive (*T. gondii* RH strain DNA), negative (DNA from pathogen-free Swiss mice), and no-template (ultra-pure water) controls to ensure assay specificity and to monitor potential contamination. Aliquots of each PCR product were separated by electrophoresis on a 1.5% agarose gel containing RedSafe Nucleic Acid Staining Solution (20,000×; iNtRON Biotechnology, Inc., Seongnam-si, Republic of Korea) and visualized under UV light using the Bio-Rad BioDoc-It™ Imaging System (Bio-Rad Laboratories, Hercules, CA, USA). DNA fragment sizes were estimated by comparison with a 100 bp DNA ladder (Meridian Bioscience, Newtown, OH, USA).

### 2.4. Statistical Analysis

Point estimates and 95% confidence intervals (95% CI) were calculated for anti-*T. gondii* antibodies and *T. gondii* DNA. Analyses were performed overall by rearing system (pet and farm rabbits), breed (small, medium, large and mixed), sex (males, females and unknown), age (juveniles and adults), vaccination status (vaccinated and unvaccinated), body condition score (2/5, 3/5 and 5/5), season of sampling (spring, summer, autumn and winter), and county of origin (Alba, Bistrița-Năsăud, Cluj, Satu-Mare and Sălaj). Prevalence differences between groups were assessed using the chi-square test. Univariate analysis and a multivariate logistic regression analysis were performed to evaluate potential risk factors associated with *T. gondii* infection. Odds ratios (ORs) and 95% CI were calculated, and a *p*-value < 0.05 was considered statistically significant. The following categories were used as reference: rearing system (pet rabbits), breed (small), sex (male), age (juveniles), vaccination status (unvaccinated), BCS (2/5), season (spring), and county (Alba). All other categories were compared with these reference groups. Data were analyzed using EpiInfo 2000 (CDC, Atlanta, GA, USA) (http://www.cdc.gov/epiinfo/index.html, accessed on 10 November 2025).

## 3. Results

The overall seroprevalence of anti-*T. gondii* antibodies in rabbits by MAT was 16.5% (58/352; 95% CI 12.9–20.7) (Table 1). The seroprevalence was higher in farm rabbits (51/304; 16.8%) than in pet rabbits (7/48; 14.6%), but not statistically significant. Statistically significant differences were observed in the body condition score category, with the highest seroprevalence detected in the 5/5 subgroup (12/36; 33.3%; *p* = 0.017). Likewise, in the seasonal categories, seroprevalence was the highest in samples collected during the summer months (16/61; 26.2%; *p* = 0.008).

Univariate analysis identified the season of sampling and county as factors associated with variation in *T. gondii* infection. Higher odds of infection were observed in summer (OR = 3.12; *p* = 0.03). Prevalence was also higher in Bistrița-Năsăud and Cluj counties. No significant associations were found for rearing system, breed, sex, age, vaccination status, or body condition score (Table 1).

A multivariate logistic regression analysis was conducted to evaluate the association between rearing system, breed, sex, age, vaccination status, BCS, season, county, and the seroprevalence of anti-*T. gondii* antibodies. Body condition score (BCS) was the only variable significantly associated with seropositivity, with higher BCS increasing the odds. Age showed a borderline association, suggesting that older animals may have lower odds of being seropositive. No significant associations were observed for rearing system, breed, sex, vaccination status, season, or county (*p* > 0.05) (Table 2).

**Figure 2:**
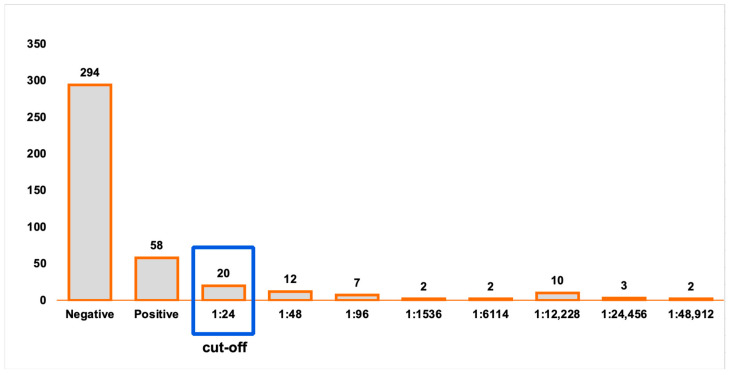
Number of seropositive samples (MAT) per dilution, starting with a cut-off of 1:24 until the endpoint 1:48,912.

The maximum endpoint titer obtained at MAT was 1:48,912. Twenty samples were positive at 1:24 dilution (34.5%), 12 at 1:48 dilution (20.7%), 7 at 1:96 dilution (12.1%), 2 at 1:1536 dilution (3.4%), 2 at 1: 6114 dilution (3.4%), 10 at 1:12,228 dilution (17.2%), 3 at 1:24,456 dilution (5.2%) and 2 at 1:48,912 dilution (3.4%) (Figure 2).

The overall prevalence of *T. gondii* DNA in rabbits was 4.1% (2/49; 95% CI 0.5–14) (Table 3). The two *T. gondii* DNA positive samples were obtained from adult (2/36; 5.5%; 95% CI 0.7–18.7) males (2/28; 7.2%; 0.9–23.5%) with a body condition score of 3/5 (2/42; 4.8; 95% CI 0.6–16.1). The rabbits that tested positive by nested PCR were also seropositive, with antibody titers of 1:12,228 and 1:48,912, respectively. Both animals were from Cluj county (2/44; 4.5%; 95% CI 0.6–15.5), one was a pet rabbit of mixed breed (1/17; 5.9%; 95% CI 0.15–27), while the other was a farm rabbit of Transylvania Giant breed (1/32; 3.1%; 95% CI 0.1–16.2).

*Toxoplasma gondii* DNA was detected in 1.5% (2/133, 95% CI 0.2–5.4) of the tested rabbit organs. The two *T. gondii* positive samples were obtained from lung and heart tissues, respectively.

## 4. Discussion

The prevalence of *T. gondii* in rabbits in Romania evaluated by MAT and nested PCR was 16.5% (58/352) and 4.1% (2/49), respectively. To the best of our knowledge, this is the first study in Romania aimed at evaluating natural *T. gondii* infection in rabbits, specifically in both pet rabbits and those raised in household farms.

In the 1960s–1970s, the seroprevalence identified in rabbits in Western Europe was higher, reaching 53% in Germany [27] and 53–57.9% in the Czech Republic [28,29]. Nowadays the seroprevalence of *T. gondii* in pet rabbits in Europe ranges from 1.41% [30] to 12.12% [31], as determined by an ELISA (enzyme-linked immunosorbent assay) and MAT, respectively, with both values reported in Poland. In Italy, a seroprevalence of 14.6% was reported in commercial rabbit farms using an indirect fluorescence antibody test (IFAT) [32]. With regard to the rearing system, a seroprevalence of 0.4% was observed in rabbits from commercial farms, whereas a higher seroprevalence (10.1%) was reported in rabbits from household farms [33]. In wild rabbits, an even higher seroprevalence of 14.2% has been reported in Spain [34]. Outside Europe, similar rates were observed in pet rabbits in Japan (0.89%) [5] and in both domestic rabbits and pet rabbits in China (10.55–13.06%) [35,36]. In contrast, higher seroprevalence rates have been reported, with IgG antibodies detected in 26.7% of domestic rabbits in Egypt [15]. In all of these studies, rabbits were tested using an ELISA.

In the present study, we identified a seroprevalence of 14.6% (7/48) in pet rabbits and 16.8% (51/304) in farm rabbits using MAT, slightly higher than rates reported in other European studies. This seropositivity may be influenced by several regional factors. In Romania, it is common to supplement rabbit diets with fresh greens from local gardens, which can increase the risk of exposure to *T. gondii* oocysts shed by stray cats. Additionally, biosecurity measures are often lacking in household facilities. The high density of definitive hosts in urban and rural areas further contributes to a greater environmental parasite load.

The modified agglutination test (MAT) is considered one of the most widely used and reliable serological methods for detecting anti-*T. gondii* antibodies in animals, particularly in epidemiological studies, due to its high sensitivity, specificity, and the fact that it does not require species-specific conjugates [1]. This method is also widely used for the serological diagnosis of *T. gondii* infection in rabbits [37,38]. In addition to MAT, ELISA, and IFAT, the latex agglutination test (LAT) can also be used to evaluate anti-*T. gondii* antibodies in rabbits [5]. Based on a comparative evaluation of tests, McKenny et al. [39] reported that MAT demonstrated good diagnostic performance, showing a sensitivity of 84.1% and a specificity of 96.7% when compared with IFAT as the reference method, with an overall agreement of 94.3%.

The relationship between MAT titers and the detection of viable *T. gondii* has also been explored [40,41]. A study conducted on sheep demonstrated a significant correlation between increasing MAT titers and the probability of isolating viable *T. gondii* through mouse bioassay. In that study, parasites were isolated in approximately 65% of cases when MAT titers were ≥1:16, whereas isolation was unsuccessful in most cases with lower titers, suggesting that higher antibody titers may reflect a greater likelihood of parasite presence in tissues. Although these findings were obtained from a different host species and biological matrix, they further support the relevance of MAT in epidemiological studies investigating *T. gondii* infection [40].

The detection of *T. gondii* genomic DNA can be performed using several techniques, including conventional PCR, nested PCR or qPCR [31,41,42]. Nested PCR targeting the B1 gene performed on pet rabbit blood samples did not yield any positive results [31]. Using the same technique, *T. gondii* DNA was detected in 2.8% of domestic rabbits. The parasite DNA was identified predominantly in brain tissue [43]. However, a study conducted on farm rabbit blood samples reported a *T. gondii* DNA prevalence of 16.2% [42].

In the present study, *T. gondii* DNA was detected in two rabbits (4.1%; 2/49). Brain, heart, liver, and lung samples from these two animals were analyzed by nested PCR. In one rabbit, *T. gondii* DNA was detected exclusively in the lung, with the rabbit being seropositive with an antibody titer of 1:12,228. In the other rabbit, DNA was detected only in the brain, and the rabbit was seropositive with an antibody titer of 1:48,912. The detection of *T. gondii* DNA may be limited to certain tissues due to the uneven distribution of tissue cysts, which can reduce PCR sensitivity [44]. Teo et al. [45] reported the presence of *T. gondii* DNA in a wider range of tissues, including the lungs, spleen, liver, femoral bone marrow, and haired skin, from two rabbits with clinical toxoplasmosis. In contrast, another study on rabbits with clinical toxoplasmosis reported isolation of *T. gondii* from brain tissue, with no detection in the liver, spleen, kidneys, lungs, heart or skeletal muscles [46].

Within the potential risk factors for *T. gondii* infection in rabbits, the following were considered: breed; age; sex; area of origin; type of feed, including the consumption of washed or unwashed vegetables; presence of cats; sampling season; health condition; and rearing system (pet versus farm rabbits) [15,31,35,47]. Among these, age (older than one year), rearing system (backyard rabbits), the presence of cats [15,48] and type of feed (mixture of fruits, vegetables or grains) [15] and unwashed vegetables were identified as significant risk factors [31]. Sex was also identified as a potential risk factor in wild rabbits, with *T. gondii* infection being significantly associated with female individuals (*p* < 0.001) [49]. In the present study, we found that rearing system, breed, age and sex were not significant risk factors, consistent with findings from previous studies [31,34]. However, body condition score (BCS) and the sampling period were both significantly associated with *T. gondii* infection, with BCS showing significance in both logistic regression (*p* = 0.02) and chi-square analysis (*p* = 0.017), indicating higher infection odds in animals with better body condition. Age showed a borderline protective effect in the logistic model, while the significant effect of the sampling period in chi-square analysis (*p* = 0.008) suggests possible temporal variations in exposure. Similar to our results, Meng et al. [47] reported a higher seroprevalence of *T. gondii* antibodies in rabbit samples collected during the summer (*p* < 0.001). Conversely, a study conducted in Australia reported no differences in *T. gondii* seroprevalence, as determined by MAT, among samples collected during summer, autumn, winter, or spring [39]. One possible explanation for the higher seroprevalence observed in summer is that temperatures during this season create favorable conditions for the sporulation of *T. gondii* oocysts, increasing the likelihood of exposure and infection in rabbits [47]. The body condition score is correlated with rabbit health, with deviations from the optimal BCS indicating higher susceptibility to disease or poorer overall health [50]. Higher *T. gondii* seroprevalence (33.3%; 12/36) in rabbits with high body condition score (5/5) may reflect age as a confounding factor, since older rabbits tend to have both higher BCS and cumulative exposure.

We acknowledge that the use of a convenience sampling approach rather than a formal randomization protocol may limit the generalizability of our findings, and the small number of PCR-positive samples (2/49) limits the statistical power of our molecular analysis. Thus, these findings should be considered preliminary, serving as a basis for future larger-scale studies. Another limitation of the present study is the small number of paired blood and tissue samples tested. Further large-scale studies involving a higher number of rabbits, as well as bioassays to allow for isolation and molecular characterization, are needed to better clarify the epidemiological role of rabbits. In this study, we attempted multiplex nested PCR-RFLP to identify the genotype in the two positive samples. However, due to the low DNA concentration, this was not successful.

## 5. Conclusions

The present study provides the first serological and molecular evidence of *T. gondii* in rabbits in Romania, with a seroprevalence of 16.5% and DNA detected in 4.1% of tissue samples. These findings enhance our understanding of the parasite’s distribution in Eastern Europe and underscore the epidemiological role of rabbits in the local transmission cycle. While the presence of the parasite in tissue samples suggests a potential route for human exposure, further large-scale studies are required to clarify public health implications. Nonetheless, these results serve as a critical baseline for future zoonotic risk assessments and highlight the importance of continued monitoring of *T. gondii* in livestock.

## Figures and Tables

**Table 1:** The seroprevalence of *T. gondii* in rabbits from Romania using MAT.

Animals	No.	Frequency	Seroprevalence % (95% CI)	OR (95% CI)	*p*
Rearing system					
Pet rabbits	48	7	14.6 (6.1–28.3)	r	
Farm rabbits	304	51	16.8 (13.1–21.4)	1.2 (0.5–2.7)	0.7
Breed					
Small	52	6	11.5 (4.4–23.4)	r	
Medium	86	11	12.8 (6.6–21.7)	1.1 (0.4–3.3)	0.8
Large	79	15	19 (11–29.4)	1.8 (0.7–5)	0.3
Mixed	135	26	19.3 (13.6–26.9)	1.8 (0.7–4.7)	0.2
Sex					
Males	166	24	14.5 (9.5–20.7)	r	
Females	179	32	17.9 (12.6–24.3)	1.3 (0.7–2.3)	0.4
Unknown	7	2	28.6 (3.7–71)	2.4 (0.4–12.7)	0.3
Age					
Juveniles ≤ 4 months old	70	15	21.4 (13.5–32.2)	r	
Adults > 4 months old	282	43	15.3 (11.3–20.2)	0.7 (0.4–1.3)	0.2
Vaccination status					
Vaccinated	188	32	17.0 (12.3–23)	1.08 (0.6–1.9)	0.8
Unvaccinated	164	26	15.9 (10.9–22.5)	r	
BCS					
2/5	7	1	14.3 (0.4–57.9)	r	
3/5	261	38	14.6 (10.7–19.5)	1 (0.1–8.7)	1
5/5	36	12	33.3 (19.3–50.3) *	2.8 (0.3–25.7)	0.4
Season of sampling					
Spring	62	6	9.7 (3.6–19.9)	r	
Summer	61	16	26.2 (15.8–39.1) *	3.1 (1.1–8.8)	0.03 *
Autumn	148	17	11.5 (6.8–17.8)	1.2 (0.4–3.3)	0.7
Winter	81	19	23.5 (14.75–34.18)	2.7 (1–7.5)	0.05
County					
Alba	66	5	7.6 (2.5–16.8)	r	
Bistrița-Năsăud	50	11	22 (11.5–36)	3.5 (1.1–11)	0.03 *
Cluj	171	33	19.3 (13.7–26)	3.1 (1.2–8.2)	0.02 *
Satu Mare	45	6	13.3 (5–26.8)	2 (0.5–6.7)	0.31
Sălaj	20	3	15 (3.2–37.9)	2.1 (0.5–9.9)	0.3
Total	352	58	16.5 (12.9–20.7)		

No. = number of rabbits; 95% CI = 95% confidence interval; OR = odds ratios; * *p* < 0.05; r = reference variable.

**Table 2:** Multivariable logistic regression analysis of factors associated with *T. gondii* seroprevalence in rabbits.

Variable	aOR (95% CI)	*p*
Rearing system	1.06 (0.44–2.58)	0.9
Breed	0.84 (0.63–1.11)	0.2
Sex	1.25 (0.72–2.16)	0.4
Age	0.48 (0.21–1.06)	0.07
Vaccination status	1.44 (0.75–2.76)	0.3
BCS	2.51 (1.15–5.51)	0.02 *
Season of sampling	1.25 (0.91–1.71)	0.2
County	0.98 (0.75–1.28)	0.9

aOR = adjusted odds ratios; 95% CI = 95% confidence interval; * *p* < 0.05; reference category: rearing system (pet rabbits), breed (small), sex (male), age (juveniles), vaccination status (unvaccinated), BCS (2/5), season (spring), and county (Alba).

**Table 3:** The prevalence of *T. gondii* DNA in rabbits from Romania using nested PCR.

Animals	No.	Frequency	Prevalence % (95% CI)	OR (95% CI)	*p*
Rearing system					
Pet rabbits	17	1	5.9 (0.15–27)	r	1
Farm rabbits	32	1	3.1 (0.1–16.2)	0.5 (0.03–8.5)	
Breed					
Small	10	0	-	r	1
Medium	6	0	-	1.5 (0.05–40.1)	1
Large	19	1	5.3 (0.1–26.5)	2.7 (0.1–70.3)	1
Mixed	14	1	7.1 (0.2–33.9)	2.4 (0.1–63.6)	1
Sex					
Males	28	2	7.2 (0.9–23.5)	r	
Females	19	0	-	0.3 (0.01–5.9)	0.49
Unknown	2	0	-	0.8 (0.02–28.5)	1
Age					
Juveniles ≤ 4 months old	13	0	-	r	
Adults > 4 months old	36	2	5.6 (0.7–18.7)	3.2 (0.1–72.7)	0.5
Vaccination status					
Vaccinated	11	1	9.1 (0.2–41.3)	3.7 (0.2–64.5)	0.4
Unvaccinated	38	1	2.6 (0.1–13.8)	r	
BCS					
2/5	3	0	-	r	
3/5	42	2	4.8 (0.6–16.1)	0.9 (0.04–21.3)	1
5/5	4	0	-	1.9 (0.1–46.9)	1
Season of sampling					
Spring	10	0	-	r	
Summer	4	0	-	1.9 (0.1–46.9)	1
Autumn	16	2	12.5 (1.5–38.3)	11.6 (0.5–25.6)	0.1
Winter	19	0	-	0.3 (0.01–6.4)	0.5
County					
Alba	5	0	-	r	
Bistrița-Năsăud	-				
Cluj	44	2	4.5 (0.6–15.5)	0.7 (0.03–15.3)	1
Satu Mare	-				
Sălaj	-				
Total	49	2	4.1 (0.5–14)		

No. = number of rabbits; 95% CI = 95% confidence interval; OR = odds ratios; *p* < 0.05; r = reference variable.

## Data Availability

The data presented in this study are available upon request from the corresponding author due to owner privacy reasons.
